# Supplementary material for: Mapping the evolution of fertility support policies in China: A content and instrumental analysis
Source: PLoS One. 2025 Oct 9;20(10):e0332137. doi: 10.1371/journal.pone.0332137 (PMC12510515; doi:10.1371/journal.pone.0332137)
Supplement: S1 Appendix — (ZIP) [file pone.0332137.s001.zip › S1 Appendix. 226 original policy documents/198-国家发展改革委、工业和信息化部、民政部等关于印发《进一步优化供给推动消费平稳增长促进形成强大国内市场的实施方案(2019年)》的通知(FBM-CLI-4-329147).doc]

国家发展改革委、工业和信息化部、民政部等关于印发《进一步优化供给推动消费平稳增长促进形成强大国内市场的实施方案(2019年)》的通知

**发布部门：** [国家发展和改革委员会(含原国家发展计划委员会、原国家计划委员会)](https://www.pkulaw.com/law/chl?Aggs.IssueDepartment=60301) [工业和信息化部](https://www.pkulaw.com/law/chl?Aggs.IssueDepartment=60234) [民政部](https://www.pkulaw.com/law/chl?Aggs.IssueDepartment=60208) [机构沿革](javascript:void(0))

**发文字号：**发改综合〔2019〕181号

**发布日期：**2019.01.28

**实施日期：**2019.01.28

**时效性：** [现行有效](https://www.pkulaw.com/law/chl?Aggs.TimelinessDic=01)

**效力级别：** [部门规范性文件](https://www.pkulaw.com/law/chl?Aggs.EffectivenessDic=XE0302)

**法规类别：** [市场管理](https://www.pkulaw.com/law/chl?Aggs.Category=08204)

国家发展改革委、工业和信息化部、民政部、财政部、住房城乡建设部、交通运输部、农业农村部、商务部、国家卫生健康委、市场监管总局关于印发《进一步优化供给推动消费平稳增长促进形成强大国内市场的实施方案（2019年）》的通知
（发改综合〔2019〕181号）

各省、自治区、直辖市及计划单列市、新疆生产建设兵团发展改革委、工业和信息化主管部门、民政厅（局）、财政厅（局）、住房城乡建设厅（规划国土委、规划局、规划国土局）、交通运输厅（局、委）、农业农村（农牧、农业）厅（局、委）、商务主管部门、卫生健康委（卫生计生委）、市场监督管理部门：
　　为贯彻落实中央经济工作会议精神，顺应居民消费升级大趋势，进一步优化供给，更好满足人民群众对美好生活的需要，促进形成强大国内市场，国家发展改革委会同有关部门共同研究制定了《进一步优化供给推动消费平稳增长 促进形成强大国内市场的实施方案（2019年）》。现印发你们，请结合本地区本部门实际，认真抓好贯彻落实。

国家发展改革委
工业和信息化部
民　　政　　部
财　　政　　部
住房城乡建设部
交 通 运 输 部
农 业 农 村 部
商　　务　　部
国家卫生健康委
市场监管总局
2019年1月28日

　　进一步优化供给推动消费平稳增长促进形成强大国内市场的实施方案（2019年）

　　为深入贯彻中央经济工作会议精神，加快落实《中共中央[国务院关于完善促进消费体制机制进一步激发居民消费潜力的若干意见](https://www.pkulaw.com/chl/50dd2ac3d611cf7dbdfb.html)》（中发〔2018〕32号）和《完善促进消费体制机制实施方案（2018-2020年）》（国办发〔2018〕93号），着力引导企业顺应居民消费升级大趋势，加快转型升级提升供给质量和水平，以高质量的供给催生创造新的市场需求，更好满足人民群众对美好生活的向往，促进形成强大国内市场，推动消费平稳增长，特制订如下方案。
　　**一、**多措并举促进汽车消费，更好满足居民出行需要
　　（一）有序推进老旧汽车报废更新。按规定放开报废汽车“五大总成”再制造再利用。有条件的地方可依托市场交易平台，对报废国三及以下排放标准汽车同时购买新车的车主，给予适当补助。对淘汰更新老旧柴油货车、推广使用新能源汽车等大气污染治理措施成效显著的地方，中央财政在安排相关资金时予以适当倾斜支持。
　　（二）持续优化新能源汽车补贴结构。坚持扶优扶强的导向，将更多补贴用于支持综合性能先进的新能源汽车销售，鼓励发展高技术水平新能源汽车。落实新能源货车差别化通行管理政策，提供通行便利，扩大通行范围。
　　（三）促进农村汽车更新换代。有条件的地方，可对农村居民报废三轮汽车，购买3.5吨及以下货车或者1.6升及以下排量乘用车，给予适当补贴，带动农村汽车消费。
　　（四）稳步推进放宽皮卡车进城限制范围。在评估河北、辽宁、河南、云南、湖北、新疆6省区放开皮卡车进城限制试点政策效果基础上，稳妥有序扩大皮卡车进城限制范围。
　　（五）加快繁荣二手车市场。进一步落实全面取消二手车限迁政策，严防限迁政策出现回潮。对二手车经销企业销售二手车，落实适用销售旧货的增值税政策，依照3%征收率减按2%征收增值税。
　　（六）进一步优化地方政府机动车管理措施。已实施汽车限购政策的地方，要结合本地实际情况，优化机动车限购管理措施，有条件的地方可适度盘活历年废弃的购车指标，更好满足居民汽车消费需求。

　　**二、**补足城镇消费供给短板，更好满足城镇化和老龄化需求
　　（七）加快推进老旧小区和老年家庭适老化改造。有条件的地方可对老旧小区加装电梯、无障碍通道、适老化家居环境、适老辅具等方面进行补贴，调动市场积极性。
　　（八）进一步满足农业转移人口市民化住房消费需求。加快发展住房租赁市场，发挥国有租赁企业对市场的引领、规范、激活和调控作用，支持专业化、机构化住房租赁企业发展。将符合条件的农业转移人口纳入住房保障范围。支持部分人口净流入、房价高、租赁需求缺口大的大中城市多渠道筹集公租房和市场租赁住房房源，将集体土地建设租赁住房作为重点支持内容。
　　（九）完善托幼等配套政策鼓励居民按政策生育。鼓励地方政府通过采取提供场地、减免租金等政策措施，加大对社会力量开展3岁以下婴幼儿托育服务的支持力度。持续促进家政服务提质扩容。
　　（十）加强城市养老设施建设提升养老服务供给水平。加大城市养老服务用地供给，加强城市闲置资源转化利用，规划建设一批、改建扩建一批养老服务机构。升级改造大城市郊区、周边地区养老服务机构，建设一批社区医养结合服务中心，增加社区护理床位供给。

　　**三、**促进农村消费提质升级，拉动城乡消费联动发展
　　（十一）着力挖掘农村网购和旅游消费潜力。充分发挥邮政系统、供销社系统和信息进村入户工程现有农村网点布局优势，引导电商企业在乡镇和农村建设服务网点，开展品牌消费、品质消费系列活动。建立健全住宿餐饮等乡村旅游和服务标准，开展休闲农业和乡村旅游精品发布推介活动。
　　（十二）持续畅通城乡双向联动销售渠道。鼓励地方特别是中西部地区和“三区三州”深度贫困地区政府，与电商企业开展多种形式的合作，挖掘优质特色农产品，加强品牌培育，集中展示销售。有条件的地方可针对本地优质特色农产品网络销售进行补贴。大力发展连锁化、品牌化便利店。确定一批重点城市开展城乡高效配送专项行动。
　　（十三）优化农村消费市场环境。加大农村流通产品监督抽查覆盖面，强化消费领域大数据在打击假冒伪劣方面的应用，扩大农村对合格产品的消费使用。积极开展有机产品认证，提升食品农产品供给质量。

　　**四、**加强引导支持，带动新品消费
　　（十四）支持绿色、智能家电销售。有条件的地方可对产业链条长、带动系数大、节能减排协同效应明显的新型绿色、智能化家电产品销售，给予消费者适当补贴。
　　（十五）促进家电产品更新换代。有条件的地方可对消费者交售旧家电（冰箱、洗衣机、空调、电视机、抽油烟机、热水器、灶具、计算机）并购买新家电产品给予适当补贴，推动高质量新产品销售。
　　（十六）积极开展消费扶贫带动贫困地区产品销售。各级机关、国有企事业单位、金融机构、大专院校等在同等条件下优先采购贫困地区产品，推动帮扶省市与贫困地区建立长期稳定的供销关系，鼓励民营企业采取“以购代捐”“以买代帮”等方式采购贫困地区产品和服务。

　　**五、**扩大优质产品和服务供给，更好满足高品质消费需求
　　（十七）打造中高端消费载体。改造提升重点城市步行街，加快国际消费中心城市培育建设。认定公布一批中华老字号品牌。抓紧出台商品交易市场发展平台经济的指导意见，促进商产融合。
　　（十八）扩大升级信息消费。加快推出5G商用牌照。支持有条件的地方建设信息消费体验中心。推动开展信息消费体验周、城市行、技能培训等各类活动，推广典型案例经验，培养信息消费习惯。
　　（十九）加快推进超高清视频产品消费。加大对中央和地方电视台4K超高清电视频道开播支持力度，丰富超高清视频内容供给。支持广电网络和电信网络升级改造，提升超高清视频传输保障能力。有条件的地方可对超高清电视、机顶盒、虚拟现实/增强现实设备等产品推广应用予以补贴，扩大超高清视频终端消费。
　　（二十）促进离境退税商品销售。优化离境退税服务网点布局，鼓励增设退税商店，研究完善退税代理机构条件，提高离境退税服务便利化水平。

　　**六、**完善政策体系，进一步优化消费市场环境
　　（二十一）持续完善消费基础设施。推进实施中小城市、农村以及边远地区、林牧区、海岛等区域基础网络完善工程，进一步为扩大和升级信息消费提供支撑。加快补齐道路、停车场、能源、电信、物流、新能源汽车充电设施等方面建设短板，推进厕所革命、垃圾和污水治理等农村人居环境整治。
　　（二十二）加强质量认证体系建设。充分发挥质量认证保障安全底线，对涉及人身健康、安全环保的产品实行强制性认证，将防爆电器、家用燃气具、500升以上大冰箱等3类产品列入强制性产品认证目录范围，推进落实强制性产品认证改革方案，促进认证制度简捷便利。鼓励企业开展自愿认证，健全认证采信机制。
　　（二十三）持续深化收入分配改革。全面实施修改后的[个人所得税法](https://www.pkulaw.com/chl/d74fa423af05cffdbdfb.html)及其实施条例，落实好专项附加扣除政策。进一步提高相对低收入群体的待遇。
　　（二十四）进一步加大消费者权益保护力度。深入开展放心消费创建活动，推动大型零售企业进行线下购物无理由退货制度试点。运用多种方式和载体，全面启动消费投诉公示工作，督促经营者诚信经营。联合开展消费者个人信息保护和预付卡治理专项行动，净化消费环境。
　　各地区各部门要充分认识优化供给、推动消费平稳增长对促进形成强大国内市场、满足人民群众日益增长的美好生活需要具有重要意义，切实强化组织领导，突出保障协调，推动政策切实落地生效。

© 北大法宝：（[www.pkulaw.com](https://www.pkulaw.com/)) 专业提供法律信息、法学知识和法律软件领域各类解决方案。北大法宝为您提供丰富的参考资料，正式引用法规条文时请与标准文本核对。
欢迎查看所有[产品和服务](https://www.pkulaw.com/helps/2.html)。法宝快讯：[如何快速找到您需要的检索结果？](https://www.pkulaw.com/helps/70.html)[法宝](https://www.pkulaw.com/helps/69.html)V6有何新特色？

扫描二维码阅读原文

**原文链接：**<https://www.pkulaw.com/chl/6caeeaf210bbedbdbdfb.html>
